# The human microbiome: A promising target for lung cancer treatment

**DOI:** 10.3389/fimmu.2023.1091165

**Published:** 2023-01-30

**Authors:** Ying Sun, Miaomiao Wen, Yue Liu, Yu Wang, Pengyu Jing, Zhongping Gu, Tao Jiang, Wenchen Wang

**Affiliations:** Department of Thoracic Surgery, The Second Affiliated Hospital, Air Force Medical University, Xi’an, China

**Keywords:** microbiome, pulmonary infection, immunology, clinical application, malignant mechanism, lung cancer

## Abstract

Lung cancer is the leading cause of cancer-related deaths worldwide, and insights into its underlying mechanisms as well as potential therapeutic strategies are urgently needed. The microbiome plays an important role in human health, and is also responsible for the initiation and progression of lung cancer through its induction of inflammatory responses and participation in immune regulation, as well as for its role in the generation of metabolic disorders and genotoxicity. Here, the distribution of human microflora along with its biological functions, the relationship between the microbiome and clinical characteristics, and the role of the microbiome in clinical treatment of lung cancer were comprehensively reviewed. This review provides a basis for the current understanding of lung cancer mechanisms with a focus on the microbiome, and contributes to future decisions on treatment management.

## 1 Introduction

Lung cancer has the highest morbidity and mortality worldwide, with approximately 2 million new cases and 1.76 million deaths in 2021 (1, 2). In recent years, researchers have found that more than 16% of cancer cases are related to infections, and most infections are caused by microorganisms (3). The relationship between microbes and cancer has attracted considerable attention in academia. Bacteria were first discovered in tumors over a hundred years ago, and the existence of microorganisms in various tumors has been successively reported (4, 5). Healthy lungs are traditionally thought to be sterile, but recent studies have found that they also harbor microbial communities, including *Firmicutes*, *Proteobacteria*, *Bacteroidetes*, and *Actinobacteria* (6, 7). In addition, early epidemiological data have suggested that bacterial infections are common in lung cancer patients, especially as the disease progresses, and it is almost 50% to 70%. The pathogenic bacteria initially colonizing the lung might persist in patients with lung cancer as the disease progresses (8). Furthermore, the microflora residing outside the lung, such as the oral cavity, airways and gut, can also affect the occurrence and development of lung cancer, suggesting that the human microflora may play a direct or indirect role in lung cancer onset and progression. This article reviews the role of the human microbiome in lung cancer as well as providing a basis for a potential role of the microbiome in therapeutic methods and drug discovery of lung cancer.

## 2 Distribution and function of human microflora

Humans coexist with and host a variety of microbes, such as bacteria, fungi, and viruses. All these microorganisms inhabiting specific areas of the human body constitute the human microbiota, which plays an important role in physiological activities such as nutrient absorption, substance metabolism, and immune regulation, and is also closely related to the occurrence of diseases such as infectious diseases, metabolic disorders, and different cancer types.

### 2.1 Distribution

#### Oral microorganisms

The oral cavity contains more than 700 species of bacteria. Oral microorganisms reside in biofilms throughout the mouth and form an ecosystem that helps to maintain a healthy microenvironment. The oral microbiota was composed of *Firmicutes*, *Bacteroidetes*, *Proteobacteria*, *Actinobacteria*, *Spirrochaetes*, and *Fusobacteria*, accounting for 94% of the total classification. The remaining phyla, such as *Saccharibacteria*, *Synergistetes*, *SR1*, *Gracilibacteria*, *Chlamydia*, *Chloroflexi*, *Tenericutes*, and *Chlorobi*, account for 6% of the taxa. The oral microbiome is impressive in its breadth and depth: one milliliter of saliva contains 1.0×10^8^ microbial cells and 700 different prokaryotic taxa. Among these, it contains bacteria, fungi, viruses, archaea, and protozoa, of which approximately 54% are culturable and have been identified, 14% are culturable and not identified, and 32% are unculturable (9).

#### Respiratory microorganisms

When the human microbial group plan was launched in 2007, the lungs were not included among the sampled organs, in part as they were thought to be sterile (10). With the increasing development and popularity of high-throughput sequencing and sequence assembly technology, together with databases of sequenced organisms (11, 12), the identification and quantification of organisms from mixed metagenomic samples has been possible through high-throughput metagenomic sequencing, a convenient, and so far the fastest strategy for the study of lung microbes (13). Respiratory microbes grow rapidly in early life of the host and are influenced by the environment, age, and immune status of the host (14). Indeed, it has been proven that birth, the first postnatal hour, and the first 3 to 4 months of exposure to the living environment are important stages for a stable development of respiratory flora (15).

In healthy lungs, two phyla are mainly present, *Bacteroidetes* and *Firmicutes*, which constitute the pulmonary microbiota, whereas *Prevotella* and *Veillonella* spp. are dominant (16–18). Compared to the upper respiratory tract, the microbiota of the lung mucosa is phylogenetically diverse. In addition, the lower respiratory system is mainly composed of *Pseudomonas*, *Streptococcus*, *Fusobacterium*, *Megacoccus* and *Sphingosphingomonas* (18, 19). Some studies have shown that the lungs are susceptible to oropharyngeal bacterial colonies (16, 17, 20). For example, Bassis et al. compared the microbial composition in the oral and nasal cavities, lungs, and stomach of healthy adults and found that the microbial communities in gastric juices and alveolar lavage fluid (BAL) were mainly derived from the inhalation and colonization of oropharyngeal flora (21).

#### Gut microorganisms

The gut provides a convenient habitat for all kinds of microorganisms, with comprise an estimated total of 1.0×10^13^ ˜ 1.0×10^14^. The human gut microbiota is composed of at least 1000 - 1200 species of bacteria, mainly *Firmicutes*, *Bacteroidetes*, *Proteobacteria*, *Actinobacteria*, *Fusobacteria*, *Verrucomicrobia*, and others. Among these, *Firmicutes* (64%) and *Bacteroidetes* (28%) were the main components in most individuals. *Actinobacteria*, *Proteobacteria*, and *Verrucomicrobia* were minor components. The human gut microbiome is extremely large and scientists have not been able to determine the number of gut microbes that people may carry. It has been estimated that a 70 kg adult (3.0×10^13^ cells) carries approximately 3.8×10^13^ bacteria (0.2 kg) (22, 23).

The composition of the human gut microbiota varies among populations, and the difference in individual composition is mainly reflected in the proportion of bacteria of each phylum. The diversity of species of gut microbiota in humans increases with time, mostly during the first three years (approximately 100 species in the first few weeks of life, 700 between six months and three years of age, and 1,000 in adulthood). Agedness is another stage at which the gut microbiota changes dramatically. At this stage, the number of facultative bacteria increases, the ratio of *Bacteroidetes* to *Firmicutes* increases, and that of *Bifidobacterium* decreases. Claesson et al. reported that, compared with young people, the differences in gut microbiota composition especially in *Ruminococcaceae* family (comprised of *Ruminococcus*, *Sporobacter*, and *Faecalibacterium* species), among individuals was significantly higher in the elderly, and the *Bifidobacterium* proportions, the *Clostridium* cluster IV, as well as the species diversity within each individual was significantly reduced, which is likely related to diet, health status, and immune system decay (24, 25).

#### Other microorganisms

Several decades ago, concentrations of intestinal bile acid were found to be much higher in breast cyst fluid than in serum in women with fibrocystic breast disease (26–28). Although the mechanisms for maintaining high bile concentrations within breast cysts remain to be studied, these studies suggest that breast tissue, like other parts of the body, is composed of an unique microbiome. Bacteria can also be detected in breast milk, possibly because microbes can travel from the surface of the skin into ducts and breast tissue. The most common genera in milk are *Staphylococcus*, *Streptococcus*, *Lactobacillus*, *Pseudomonas*, *Bifidobacterium*, *Corynebacterium*, *Enterococcus*, *Acinetobacter*, *Rothia*, *Cutibacterium*, *Veillonella* and *Bacteroides* (29). Urbaniak et al. studied the differences in microbial communities in 81 pairs of patients with and without breast cancer, and found that *Proteobacteria* composition was different between them, together with regional divergence (30).

**Figure 1:**
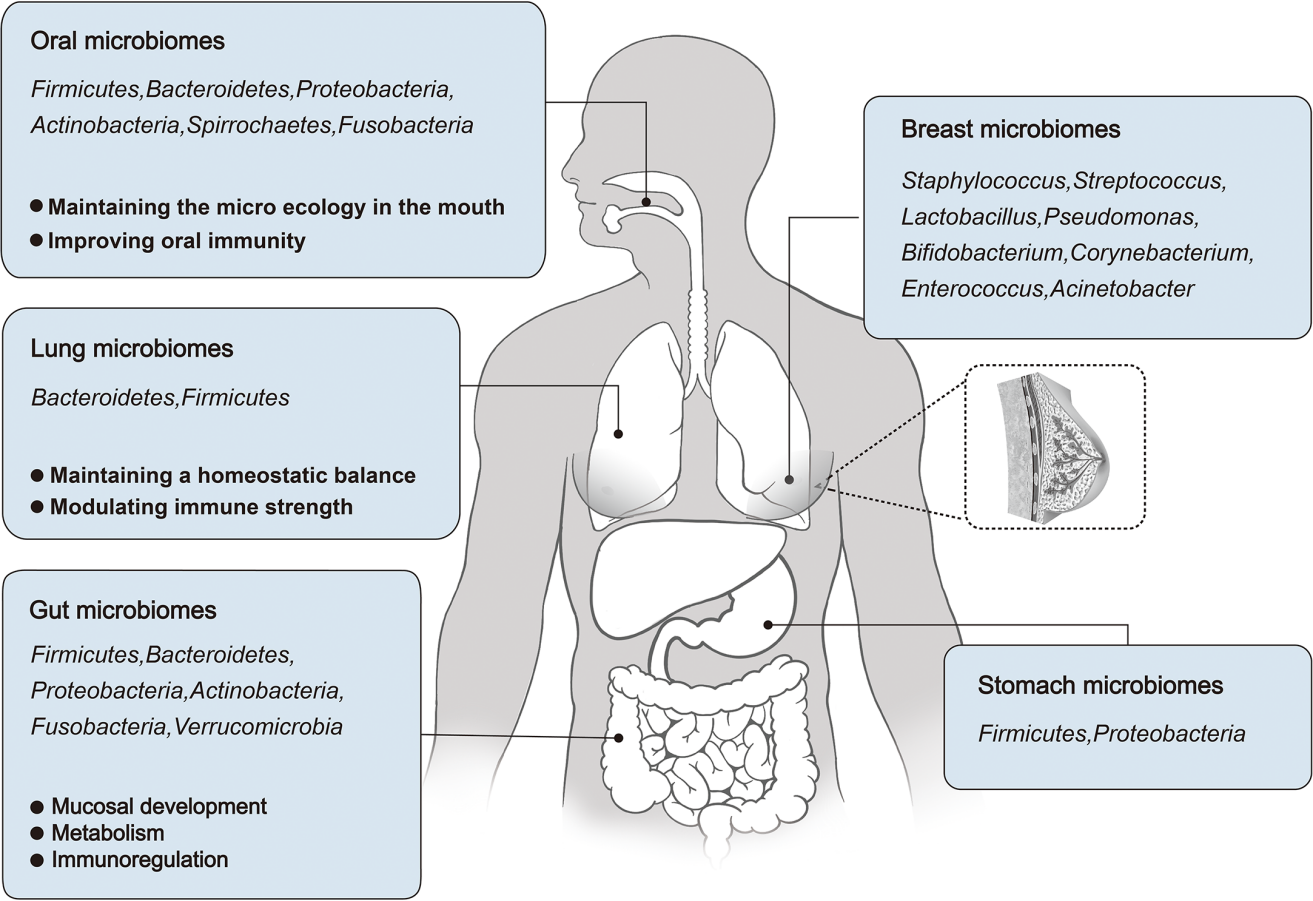
An overview of the microbial distribution in human body, and the roles of Oral, Lung, and Gut microbiomes in human development and physiological function.

The stomach has generally been considered to have fewer symbiotic bacteria because of its highly acidic environment and high protein hydrolase content (31, 32). However, recent studies have shown that a wide variety of bacteria can be found in the human stomach. *Firmicutes* and *Proteobacteria* are the major phyla, and *Streptococcus* and *Prevotella* are the major species in the stomach of individuals without *Helicobacter pylori* (HB) (33, 34) (**Figure 1**). Infection with HB can disturb the microbial community in the stomach (35).

In normal prostate, estimations concerning the microorganism number and composition are difficult since access to non-diseased prostate tissue is restricted. However, a number of previous studies have characterized the microbial composition in prostate cancer and normal surgically resected specimens, and found that no bacteria were present in normal prostate tissue (36–38). On the contrary, one study detected a positive result for bacteria in tissue specimens of benign prostatic hyperplasia (BPH) (39). However, it cannot be discarded that the positive result may owe to contamination (40). In addition, normal prostatic fluid may prevent microbial growth because of its highly antibacterial properties. Microbial invasion occurs only in the prostate upon prostatitis or other pathological occurrences (41).

### 2.2 Influence of microbiome on human development and physiological function

The microbiome plays an important role in human development and physiology. In this context, changes in the oral microbiome may cause oral and systemic diseases (42) and an imbalance in the respiratory microbiome may affect the occurrence of lung diseases (43). The gut microbiome accounts for a relatively high proportion of the human body, and its functions have been fully studied, including nutrient metabolism and immune regulation. The following sections focus on the role of oral, respiratory, and intestinal microbiota in human development and physiological function (**Figure 1**).

#### 2.2.1 Oral microbiome

The human oral microecosystem contains a large diversity of microorganisms, including bacteria, fungi, viruses, mycoplasma and protozoa. Of these, bacteria (about 700 species) make up the majority of the healthy oral microbiome and are mainly composed of six phyla, including Firmicutes, Actinobacteria, Proteobacteria, Fusobacteria, Bacteroides and Spirochaetas (44). In addition to bacteria, about 100 fungi also make up an important part of the oral microbiome, of which *Candida* is the most common. In the oral microecosystem, microbes such as bacteria and fungi attach to the surface of teeth and form a biofilm called plaque with the surrounding extracellular matrix in order to protect themselves from fluctuations in the oral environment and external drug stimuli and evade host defense mechanisms (45).

The balance of oral microecosystem not only contributes to the maintenance of oral health, but also has a potential impact on the overall health. Microorganisms in oral microecosystems achieve dynamic balance between each other and the host through complex interspecific interactions such as symbiosis, competition and confrontation (46). This paper summarizes the physiological function of normal microbial flora in oral cavity.

##### 2.2.1.1 Maintaining the microecology in the mouth

The normal microflora in oral cavity can maintain the microecological balance well. When pathogenic bacteria such as *P. seudomonas aeruginosa* invade, the oral flora inhibits their growth in saliva by producing lactic acid (47). Therefore, the normal oral flora plays an important role in preventing the invasion of pathogenic bacteria. However, disturbances in oral microecology such as oral flora imbalance or reduction of oral symbiotic bacteria provide opportunities for the invasion and colonization of respiratory pathogens such as *Staphylococcus aureus*, *Pseudococcus aeruginosa*, *Enterococcus faecalis* and *Acinetobacter* (48–51).

##### 2.2.1.2 Improving oral immunity

Natural aging, hypoplasia of parotid and submandibular glands, and medications (antihypertensive drugs, anticholinergics) can alter saliva composition or affect saliva secretion or flow rate, leading to dry mouth and poor oral hygiene (52). This may lead to the transfer of normal oral flora to communities containing more pathogens (53).

#### 2.2.2 Respiratory microbiome

The respiratory tract is a complex organ system whose main function is the exchange of oxygen and carbon dioxide. It is divided into the upper respiratory tract, which includes the nasal passages, pharynx, larynx, and lower respiratory tract, which includes the conducting airways (trachea and bronchi), small airways (bronchioles), and respiratory areas (alveoli). Because the respiratory tract is connected to the outside world, a large number of airborne microorganisms and particles, including viruses, bacteria and fungi, continue to migrate or be removed from the respiratory tract. The bacterial burden of the upper respiratory tract is about 100-10000 times than that of the lower respiratory tract, and the nasal cavity is dominated by *Propionibacterium*, *Corynebacterium*, *Staphylococcus* and *Moraxella*. *Prevotella*, *Vermicelli*, *Streptococcus*, *Haemophilus*, *Fusobacterium*, *Neisseria* and *Corynebacterium* were predominant in oral cavity (54, 55). *Prevotella*, *Vermicelli*, and *Streptococcus* colonize in the lower respiratory tract, and these microbial compositions differ from those observed in the oral and nasal cavities (56). As mentioned earlier, the gut microbiome of young children stabilizes at about 3 years of age, similar to that of adults, and this pattern of community maturation is reproduced in the upper respiratory tract microbiome (14, 57, 58). The following is a comprehensive summary of the physiological function of respiratory microorganisms in human body.

##### 2.2.2.1 Maintaining a homeostatic balance

The respiratory tract is the main site of continuous contact with exogenous microorganisms. Airway epithelium acts as a sensor for the presence of microorganisms, and its epithelial cells are in constant contact with the environment. This interaction is a key factor in maintaining stable homeostasis. The environmental conditions necessary for microbial growth in the respiratory tract (such as PH, temperature, nutrition, oxygen tension, and activation of inflammatory cells in the host) are heterogeneous, so considerable regional variation can be observed in a single healthy lung (59).

##### 2.2.2.2 Modulating immune strength

In health conditions, the microbiome can also regulate immune strength. Symbiotic fungi have been shown to influence the immune system and regulate the bacterial community, thus contributing to the recovery of bacterial flora after antibiotic treatment (60, 61).

#### 2.2.3 Gut microbiome

##### 2.2.3.1 Mucosal development

Gut microorganisms can affect intestinal mucosal development and homeostasis. Comparative studies of conventional and germ-free animals have shown that the gut microbiota is essential for the formation and functional realization of the intestinal mucosal immune system during infancy (62). A poor development of villous capillaries in the infancy of sterile mice and a consequential still dysplasia in adulthood confirmed that the gut microbiota contributes to the formation of the intestinal immune ultrastructure (63). The gut microbiome also contributes to the development of intestinal intraepithelial lymphocytes (IILs). Compared with conventionally grown animals, the production of intestinal mucosal-associated lymphoid tissue and antibodies was strongly reduced, and the original center, cell lamina propria, and cell lymphoid follicles of the mesenteric lymph node were significantly decreased in germ-free animals. Meanwhile, gut microbiota plays an important immunomodulatory role in intestinal mucosal homeostasis, with direct consequences in human health (64, 65).

##### 2.2.3.2 Metabolic

Gut microbiota improves nutrient metabolism. The gut is an important site of digestion and absorption in the human body. Here, gut microbiota can contribute in foor digestion and decomposition, also could promote intestinal peristalsis and inhibit the proliferation of pathogenic bacteria. Gut microbiota can also provide various substrates, enzymes, and energy necessary for human metabolism, and participate in metabolic processes. Among them, *Firmicutes*, *Bacteroidetes*, and some anaerobic microorganisms can decompose complex carbohydrates in the gut to produce short-chain fatty acids (SCFAs), such as acetic acid, propionic acid, and butyric acid (65–68). SCFAs are not only the energy source of gut microorganisms themselves and the intestinal epithelial cells of the host, participating in adipogenesis and gluconeogenesis, but can also regulate the intestinal immunity of the host, reducing the pH of the colonic environment and inhibiting harmful bacterial growth and colonic inflammation (69).

Most pathogens cannot compete with the resident microbiome for carbohydrate food sources and are therefore effectively excluded from the gut under normal circumstances. Thus, disruption of the gut ecosystem appears to play an important role in the establishment of pathogenic bacteria. For example, antibiotic treatment disrupts the cross-feeding network between mucinous and non-mucinous degradants and allows for pathogenic bacteria such as *Salmonella typhimurium* and *Clostridioides difficile* (70).

Besides playing a role in carbohydrate metabolism, gut microorganisms also participate in bile acid metabolism, tryptophan metabolism and other processes. Bile acids are produced in the liver and metabolized by enzymes produced by gut bacteria and are essential for maintaining a healthy gut microbiome, balancing lipid and carbohydrate metabolism, as well as innate immunity. The ability of intestinal flora to convert intestinal bile acid organisms into their unbound forms is critical to gastrointestinal .metabolic homeostasis, and these unbound bile acids activate bile acid signaling receptors (71, 72). The main bacterial genera involved in bile acid metabolism are *Bacteroides*, *Clostridium*, *Lactobacillus*, *bifidobacterium* and *listeria* (73, 74). Clinical patients with hepatoenteric diseases often present with intestinal ecological disorders characterized by reduced microbial diversity and a reduced abundance of firmicutes, leading to lower levels of intestinal secondary bile acids and higher levels of conjugated bile acids (75–77). Therefore, bile acid metabolism and intestinal flora interact, and when this balance is disrupted, a variety of clinical disease phenotypes can result.

Tryptophan metabolism is another important function of intestinal microorganisms to promote nutrient metabolism. As a nutrient enhancer, tryptophan plays a crucial role in the balance between intestinal immune tolerance and intestinal flora maintenance. Tryptophan is absorbed in the small intestine, but when it reaches the colon it can be broken down by gut bacteria such as *Clostridium sporogenes*, *Escherichia coli* and *Lactobacillus* to produce various indole derivatives that play an important role in key aspects of bacterial ecological balance (78–80).

##### 2.2.3.3 Immune regulation

Gut microorganisms regulate the human immune system through immune cells and their metabolites. Recent studies have shown that gut microorganisms can over-activate CD8+T cells, which can promote chronic inflammation and T-cell failure (81, 82). Signals from gut microbes also provide appropriate conditions for dendritic cell generation (83). Gut microorganisms can also participate in immune regulation through metabolites, which further guide or influence immune cells. For example, lactic acid and pyruvate, metabolites derived from gut microorganisms, can promote immune responses by inducing G-protein coupled receptor (GPR)-31 to mediate the production of intestinal C-X3-C Motif Chemokine Receptor (CX3CR)-1-positive dendritic cells (84). Furthermore, *Odoribacter splanchnicus* and *Bilophila* genus were negatively correlated with tumor necrosis factor (TNF)-α production following lipopolysaccharide (LPS) and *C. albicans* stimulation. *Barnesiella* was negatively associated with LPS-and *B. fragilis*-induced interferon (IFN)-γ production. This included common gut commensals, such as *Dorea longicatena* and *Dorea formicigenerans*, where higher species abundance was associated with higher IFN-γ levels in response to *C. albicans* hyphae. Both species of *Dorea* can metabolize sialic acids, which are usually found at the end of mucins; and the release of these acids is associated with mucin degradation, and may increase gut permeability. Both *Streptococcus parasanguinis* and *Streptococcus australis* were associated with IFN-γ production whereas other species, such as *Streptococcus mitis/oralis/pneumoniae*, were associated with IL-1β production. Also the correlation of *Bifidobacterium pseudocatenulatum* and IFN-γ was positive. In contrast, the correlation of *Bifidobacterium adolescentis* and TNF-α was negatively. In addition, *P. distasonis* was negatively associated with TNF-α and IL-1β after stimulation with *C. albicans* hyphae (85–88).

## 3 Relationship between microbiome and clinical features of lung cancer

### 3.1 Pathological types

The microbiota may be specifically related to the pathological types of lung cancer tissues (Details in **Table 1**). Based on histological features, lung cancer can be divided into small cell lung cancer (SCLC) and non-small cell lung cancer (NSCLC), which can be further divided into adenocarcinoma (AC), squamous cell carcinoma (SCC), and large cell carcinoma (LCC). *Klebsiella*, *Acidovorax*, *Polarmonas, and Rhodoferax* have found to be more frequent in SCLC (89). This was later confirmed by Greathouse et al. (90). *Xylobacter*, *Eufluobacter*, and *Clostridium* were also positively correlated with SCLC occurrence. However, *Prevotella* and *Pseudobutyrivibrio ruminis* may be negatively correlated with SCLC (91).

**Table 1:** Relationship between microbiome and clinical features of lung cancer.

Clinical features		Related bacteria	Potential biological functions	References
Clinical Features	SCLC	*Klebsiella, Acidovorax*, *Polarmonas*, *Rhodoferax*	More common in SCLC	(56)(57)
		*Xylobacter*, *Eufluobacter*, *Clostridium*	Be associated with the occurrence of SCLC	(58)
	NSCLC	Five genera of bacteria	Early sputum detection markers	(59)
		*Prevotella*, *Lactobacillus*, *Rikenellaceae*, *Treptococcus*, *Enterobacteriacea*, *Oscillospira*, *Bacteroides plebeius*	Fecal markers	(60)
		Leptum, Faecalibacterium prausnitzii, Ruminococcus, Clostridia	Dysregulation of butyrate metabolism	(61)
	SCC	*Acidovorax*	Enriched in squamous cell carcinoma with TP53 mutation	(57)
		*Acidovorax*, *Veillonella*	Sputum biomarkers in SCC	(59)(62)
		Microorganisms of the family Enterobacteriaceae	Be related with SCC	(63)
	AC	*Capnocytophaga*, *Selenomonas*, *Veillonella*, *Neisseria*	Biomarkers of sputum diagnostic	(59)(62)
		*Thermus*	High phylogenetic diversity	(65)
		Pseudomonas	Specific microorganisms present in adenocarcinoma	(56)
Progression and prognosis of lung cancer	Progression	*Legionella*	With higher abundance in lung cancer patients with metastasis	(65)
		*Phascolarctobacterium, Dialister*	*Phascolarctobacterium* was enriched in patients with clinical benefit, *Dialister* is more common in patients with progressive disease	(67)
		Streptococcus,	There are differences between patients with metastatic and non-metastatic NSCLC	(68)
		Veillonella, Rothia		
	Prognosis	*Koribacteraceae*	Associated with increased RFS and DFS in lung cancer patients	(70)
		*Bacteroidaceae*, *Lachnospiraceae*, *Ruminococcaceae*	Associated with reduced RFS or DFS	

The microbiome can be also used as a biomarker for NSCLC screening. Five bacterial genera showed abnormal abundance in the sputum of patients with NSCLC compared to that of controls (92). Also, contents of *Prevotella*, *Lactobacillus*, *Rikenellaceae*, *Treptococcus*, *Enterobacteriaceae*, *Oscillospira*, and *Bacteroides plebeius* were significantly higher in the feces of patients with NSCLC than in healthy controls (93). However, *Leptum*, *Faecalibacterium prausnitzii*, *Ruminococcus*, and *Clostridia* contents were found decreased in patients with NSCLC (94).

Furthermore, there were differences between the microbiomes of patients with SCC and AC. *Acidovorax* is enriched in SCC with TP53 mutations, but not in AC (90). Significant changes were observed in *Capnocytophaga*, *Selenomonas*, *Veillonella*, and *Neisseria* in SCC and AC saliva samples, whereas the microbiome of patients with SCC seemed to be more diverse than that of those with AC. Therefore, *Acidovorax* and *Veillonella* can be used as sputum biomarkers for SCC diagnosis (92) (95). SCC is specifically associated with *Enterobacteriaceae* microorganisms (96) (97). Levels of *Capnocytophaga* and *Rothia* were also higher in SCC than in AC. However, increases in *Capnocytophaga*, *Selenomonas*, *Veillonella*, and *Neisseria* were associated with AC (95). *Capnocytophaga* can be used as a diagnostic biomarker for AC sputum with 72% sensitivity and 85% specificity (92). In addition, Yu et al. observed an increased abundance of *Thermus* sp. and a decrease in the abundance of *Ralstonia* sp. In AC (98), whereas Greathouse et al. confirmed that *Pseudomonas* is specifically present in AC (89). In addition, John Cunningham (JC) virus was observed in tumor tissues and metastatic lymph nodes of patients with AC, suggesting that this virus may be involved in the occurrence of AC (99). Last, Huang et al. found that the number of *Veillonella*, *Megacoccus*, *Actinomyces* and *Arthrobacter* was significantly higher in AC than in SCC.

### 3.2 Progression and prognosis

The microbiome features are closely associated with the progression of lung cancer (**Table 1**). In this line, Guo et al. found that *Legionella* was more abundant in patients with metastatic lung cancer (98). Also, *Phascolarctobacterium* has been found to be enriched in patients with clinical benefit and has been related to an extension of progression-free survival (PFS), whereas *Dialister* is more common in patients with progressive disease, and its higher abundance is related to reduction of progression-free and overall survival (OS) (100).

Huang et al. (101) sequenced 33 cases of broncholavage fluid (14 cases of squamous cell carcinoma and 19 cases of adenocarcinoma) and 52 cases of sputum samples (15 cases of squamous cell carcinoma and 37 cases of adenocarcinoma). The results showed that the number of *Veillonell*, *Megasphaera*, *Actinomyces* and *Arthrobacter* in lung adenocarcinoma without metastasis was significantly higher than that in lung squamous cell carcinoma without metastasis. The contents of *Capnocytophaga* and *Rothia* in metastatic lung adenocarcinoma were significantly lower than those in metastatic lung squamous cell carcinoma. *Streptococcus* content was significantly lower in lung adenocarcinoma with metastasis than in lung adenocarcinoma without metastasis. The contents of *Veillonella* and *Rothia* in lung squamous cell carcinoma with metastasis were significantly higher than those in lung squamous cell carcinoma without metastasis. Jungnickel et al. (102) found that the number and volume of metastatic cancer nodules in the lung of mice exposed to *Haemophilus paraininfluenzae* increased significantly. It is speculated that *Haemophilus paraininfluenzae* may promote the upregulation of TLR2 or TLR4, induce the high expression of cytokine IL-17C, aggravate the inflammatory response of neutrophils and thus play a role in promoting cancer. In basic experiments (102, 103), it was found that *Hemophilus paraininfluenzae* in the lung and the imbalance of lung flora promoted the metastasis of mouse cancer cells to the lung, indicating that lung flora was involved in the metastasis of lung cancer. Besides, the lung and gut microbiota may affect the prognosis of patients with lung cancer (104). The potential relationship between the lung microbiome and prognosis of lung cancer has been first demonstrated by Peters. Specifically, the abundance of *Koribacteraceae* in lung tissue is associated with an increase in relapse-free survival (RFS) and disease-free survival (DFS) in patients with lung cancer. On the contrary, the abundance of *Bacteroidaceae*, *Lachnospiraceae*, and *Ruminococcaceae* was correlated with a decrease in RFS or DFS of lung cancer (105). These further indicated that the dynamic changes of some microflora might be related to the progression of lung cancer.

## 4 Microbiome and biological function of lung cancer

The human microbiome significantly affects the occurrence and development of lung cancer by regulating tumor cells and the microenvironment (106–108).

### 4.1 Tumor cells

**Figure 2:**
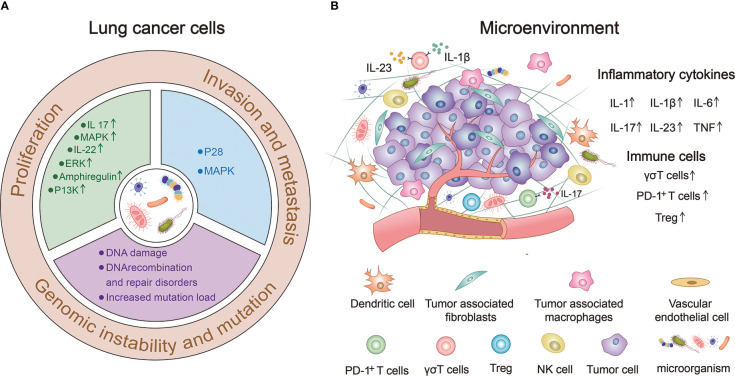
The human microbiome can significantly influence the occurrence and progression of lung cancer. **(A)** Human microbiota can directly or indirectly affect the proliferation, invasion, metastasis, genomic instability and mutation of lung cancer cells. **(B)** Human microorganisms participate in the composition of lung cancer microenvironment (TME) and regulate the occurrence and development of lung cancer by up-regulating the expression of immune cells and inflammatory factors.

Proliferation, invasion, and metastasis are the core biological characteristics of tumor cells (109). The human microbiome can directly or indirectly affect lung cancer cell proliferation, invasion, metastasis, genomic instability, and mutations (**Figure 2A**).

Enrichment of lower airway microbiota and oral symbiotic bacteria frequently occurs in lung cancer, and these bacteria can trigger the host transcriptome associated with carcinogenesis. Compared with healthy people, extracellular signal-regulated kinase (ERK)- and phosphoinositide 3-kinase (PI3K)-signaling pathways of the lower airway transcriptome in patients with lung cancer are significantly upregulated, which is related to the enrichment of *Streptococcus*, *Prevotella*, and *Veillonella* oral groups in lower airways (110). Recent studies further found that tiny *Vibrio* is the most abundant microbe that drives the upregulation of interleukin (IL)-17, PI3K, mitogen-activated protein kinase (MAPK), and ERK pathways in the airway transcriptomes of patients with lung cancer and is associated with poor prognosis (111). In human lung cancer, not only is the pulmonary microflora changed, but the local adaptive immune gamma-delta (γδ)-T cells are also activated and directly promote the proliferation of tumor cells through effector molecules such as IL-22 and amphiregulin (103). In addition to the lung microbiota, other bacteria such as HP and its produced urease may also play an important role in lung mucosal proliferation and carcinogenesis. Recently, HP urease was found to enter the lung through gastroesophageal reflux and provide an antigenic trigger for pulmonary granuloma, which leads to subsequent lung mucosal proliferation and carcinogenesis (112).

Changes in the microbiota of patients with lung cancer may contribute to advancing disease progression. The “transition” of microorganisms to *Firmicutes* in the lower lobe of the lung may be a sign of increased pathogenicity and is associated with poorer prognosis (113). Such low airway microbiota is more common in stage IIIB - IV lung cancer with lymph node metastasis (111). In addition, the gut microbiota plays an important role in the invasion and metastasis of lung cancer. Toll-like receptors (TLRs) on the membrane surface of intestinal epithelial cells are pathogen-related recognition receptors that bind different microbial ligands, such as LPS, viral double-stranded RNA, and parasites and fungi-derived toxins (114). These enter the lungs and activate the adaptive immunity through TLRs, leading to T-cell differentiation and macrophage and dendritic cell activation. For example, TLR4 stimulation by heat-inactivated *Escherichia coli* increase the adhesion, migration and metastatic diffusion of NSCLC cells *in vivo*, mainly through p38 MAPK and ERK1/2 signaling pathways (115).

Microorganisms and their metabolites may produce tumorigenic effects by directly affecting epithelial cells or oncogenes (116). Pulmonary PAH-degrading bacteria, such as *Massilia* and *Acidovorax*, are more prevalent in smokers with lung cancer and TP53 mutations. The enrichment of these bacteria is combined with the trend of DNA recombination and repair pathway disorders, suggesting that contact of lung symbiotic microorganisms with tobacco may lead to mutations in host genes (117). An imbalance in the composition of microbial flora produces various toxins that lead to genotoxicity, promote the generation of free radicals, and cause DNA damage, thereby leading to a cycle arrest and apoptosis of cells without DNA repair systems (112). In addition, other microorganisms and their metabolites, such as HP, intestinal deoxycholic acid and shicholic acid, can cause DNA damage and increase the gene mutation load, thus inducing lung cancer (112, 118).

### 4.2 Tumor microenvironment

The tumor microenvironment (TME) is an environment composed of various physical and chemical factors surrounding tumor cells, including neighbor tumor cells, immune cells, stromal cells, extracellular matrix, and a variety of soluble molecules, and is an important aspect of the tumor. TME plays an important role in the occurrence and development of tumors (119). **Figure 2B** provides a good summary of the microbial involvement in the composition of lung TME and the mechanism of regulating the occurrence and development of lung cancer (120–122).

In a mouse model of KRAS-TP53 co-mutation (KP) lung cancer, airway microbiosis disorder caused by Tiny Vibrio led to the recruitment of Th17 cells, increased IL-17 production, increased PD-1+T cell-expression, and recruitment of neutrophils, which resulted in a reduced survival and increased the burden of lung tumors (111). Gut microbiota can also activate B cells, T cells, and other immune cells, which inflate the lungs through hemato-vascular or lymphatic pathways and activate the immune response to affect lung inflammation (114, 123–125). It has been reported that an imbalance in intestinal flora may regulate the TLR4/NF-KB signaling pathway of the lung immune system by modulating the intestinal barrier, activating pulmonary oxidative stress, and mediating the response to lung injury (126). Intestinal symbiotic bacteria and their metabolites, short-chain fatty acids (SCFAs), such as propionic acid and butyric acid in patients with NSCLC directly stimulate intestinal-epithelial cells to regulate the release of T-regulatory (Treg) cells (127). Treg cells can inhibit airway inflammation by stimulating SCFAs, suggesting that immune cells play an important role in microbial-mediated inflammation (125). In addition, HP produces some relevant adaptive immune effects on T cells, in addition to inducing extensive innate immune signal transduction effects in the lungs (128, 129).

Studies have shown correlations between lung cancer cell growth and unbalances in the airway microbial community. This locally dysregulated microbiome stimulates the production of IL-1β and IL-23 in myeloid cells, which in turn induce the proliferation and adaptive activation of lung-resident Vγ6+Vδ1+γδT-immune cells. Activated γδT cells produce IL-17, which promotes neutrophil infiltration and inflammation in the TME (103, 108). The theory of IL-17-mediated inflammatory pathway has also been confirmed in other studies and animal models (130, 131). Therefore, IL-17 produced by adaptive immune γδT cells plays a role in mediating the inflammatory pathways. In addition, increasing evidence suggests that HP contributes to inducing lung tumors. HP-derived LPS induces the production of pro-inflammatory factors, including IL-1, IL-6, and TNF. This inflammation can develop into chronic bronchitis which can be often accompanied by lung cancer (132).

## 5 Research and application of microbiome in the treatment of lung cancer

**Figure 3:**
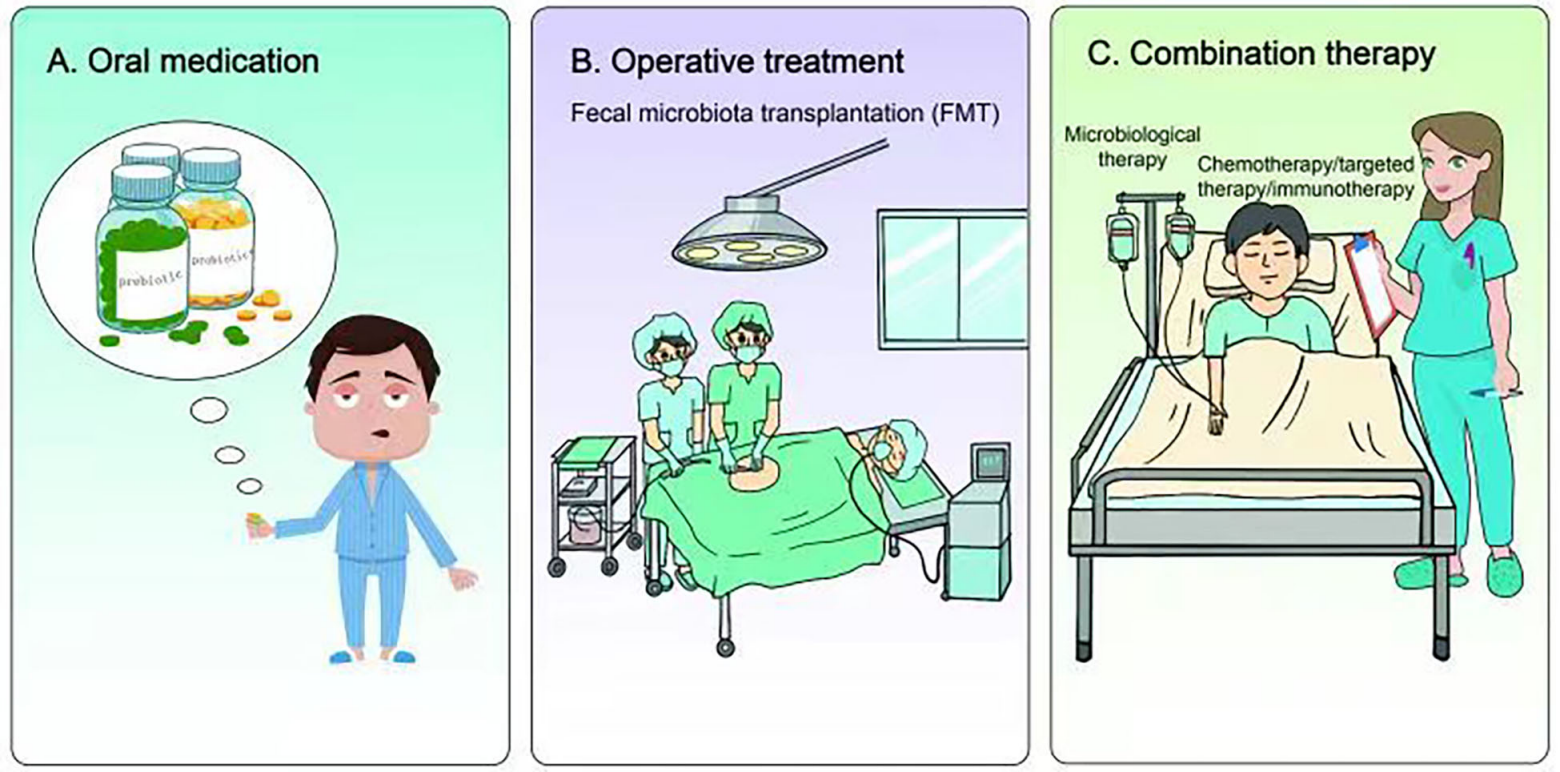
Research and application of microbiome in lung cancer treatment. **(A)** Nutritional intervention with prebiotics and probiotics can not only restore the homeostasis of internal organs or lower airway, but also reduce microbial-induced inflammation, genotoxicity and cell proliferation, thus improving the treatment of lung cancer. **(B)** Fecal microbiota transplantation (FMT) can also restore host homeostasis and reduce microbial-induced inflammation. Preclinical studies have shown that FMT therapy may have certain advantages in combating immunotherapy resistance in lung cancer. **(C)** Chemotherapy, targeted therapy or immunotherapy combined with microbial therapy can improve the clinical treatment effect of lung cancer patients.

Currently, the application of microbiomal knowledge to clinical research is a matter of extensive research. From the perspective of nutritional intervention, prebiotics and probiotics play indispensable roles. They can not only restore homeostasis of visceral organs or lower airways but also reduce microbial-induced inflammation, genotoxicity, and cell proliferation (133, 134) (**Figure 3**). Lee et al. found that *Bifidobacterium* was abundant in the intestinal tract of patients with NSCLC who responded to clinical treatment. Further, when a commercial *Bifidobacterium* strain was used to treat mice tumors with the same genotype the tumor load could be reduced by inducing the host immune response and cooperating with immunotherapeutic or chemotherapeutic drugs (135). Yusuke Tomita et al. used 588 strains of *Clostridium butyricum* (MIYAIRI 588 strain) to ameliorate symptoms associated with ecological disturbance caused by antibiotics (ATBs), suggesting that probiotic Clostridium butyricum therapy (CBT) has a positive effect on improving immune checkpoint blockade (ICB) in patients with cancer (136). On the other hand, oral administration of *Lactobacillus acidophilus* enhanced the antitumor effect of cisplatin, reduced tumor size, and improved the survival rate of mice (137). Therefore, prebiotics and probiotics can improve lung cancer treatment.

In addition to nutritional intervention of prebiotics and probiotics, fecal microbiota transplantation (FMT) also restored host homeostasis and reduced microbial-induced inflammation (138, 139) (**Figure 3**). Although there is currently a lack of clinical application of FMT in lung cancer or other tumor types, previous preclinical studies have found that FMT could reverse the response to immunotherapy of drug-resistant patients by increasing the recruitment of CCR9+CXCR3+CD4+ T lymphocytes into tumor lesions in mice. These results indicate that FMT may have some advantages in battling resistance to lung cancer immunotherapy.

A number of studies have found that the gut microbiome of patients with lung cancer who respond to clinical treatment is significantly different from that of patients who do not respond, indicating that some favorable/unfavorable microorganisms are enriched in responders and non-responders respectively, thus implying a potentially predictive value for lung cancer clinical treatment (140–142) (**Figure 3**). Concerning chemotherapy, patients with advanced lung cancer treated with *Enterococcus* and *Human Bariniella* combined with immunochemotherapy showed longer PFS (143). In terms of targeted therapy, the role and therapeutic effects of the microbiota are very optimistic according to preclinical studies (144). In a mouse lung cancer model, *Bacteroides ovatus* and *Bacteroides xylanisolvens* were positively correlated with the treatment results. Oral or intragastric administration of these responsive bacteria could significantly improve the efficacy of Erlotinib and induce CXCL9 and IFN-γ expression (144). In immunotherapy, combined microbial therapy can improve the response to and effect of immune checkpoint inhibitors (ICIs). A recent study explored the role of gut microbes in the effectiveness of immunotherapy (145). The intestinal microbial community can affect the immune regulation mechanism by regulating T cell differentiation and significantly improve the therapeutic effect of ICI (140, 146–149). Mice using stool samples from patients who responded positively to immunotherapy, whereas mice using stool samples from patients who did not respond did not. A retrospective study reported that *Clostridium butyricum* treatment (CBT) before or after ICI treatment significantly extended patients’ progression-free survival (PFS)non-progressive survival and overall survival (OS) (136). Improved survival in these patients can be attributed to more efficient immunomodulatory effects.

## 6 Future perspectives

The microbiome characteristics have significant effects in tumor development, however, how the microbiome responds to lung cancer, in particular, how lung cancer cells and TME shape the local microbial community of the lungs, is unknown. However, it has been shown that in colorectal cancer (CRC), loss of surface barrier function can cause tumor inflammation induced by symbiotic bacteria. In particular, the breakdown of tight connections between colon tumor cells allows bacterial degradation products such as LPS, to enter the tumor stroma, causing bone marrow-derived cells to be recruited to the TME. Therefore, understanding the interaction between the human microbiome and lung cancer cells, and identifying the cellular and molecular mediators involved in this interaction are relevant issues to be explored in order to find future potential targets for lung cancer treatment.

In addition, when considering the influence of microbiome on the efficacy of chemotherapy, targeted therapy, and immunotherapy for lung cancer, it is necessary to distinguish between the specific roles of the local lung microbiome, the distal gut microbiome, and oral bacteria in tumor growth and related immune responses (111). It is possible that selectively targeting one of these compartments may lead to different effects on lung cancer progression and treatment, thus providing new strategies for lung cancer treatments in the future.

## Author contributions

Both authors contributed equally to the writing of the review.
